# Effect of relative humidity, storage days, and packaging on pecan kernel texture: Analyses and modeling

**DOI:** 10.1111/jtxs.12723

**Published:** 2022-10-03

**Authors:** Himanshu Prabhakar, William L. Kerr, Clive H. Bock, Fanbin Kong

**Affiliations:** ^1^ Department of Food Science & Technology University of Georgia Athens Georgia USA; ^2^ Fruit and Tree Nut Research USDA‐ARS‐SEFNTRL Byron Georgia USA

**Keywords:** nut, physical, quality, shell, TPA, US

## Abstract

The studies expounding on the effects of storage conditions on texture changes are limited. The researchers have been proposing methods to measure pecan texture instrumentally. But current protocols and/or attributes fail to address huge variability during experimentation. Additionally, there are no predictive models to estimate changes in pecan texture during storage. This study addresses all the above concerns and investigates the effects of different relative humidity (RH, 30–90%) and packaging material (Polyethylene‐Nylon [PEN], polypropylene [PP], low density polyethylene [LDPE], and metallic laminates [ML]) on pecan texture, introducing a *rift* ratio (F/H or fracturability to hardness ratio) to address variability in the data and predictive model to estimate changes in the textural attribute of pecans during storage. The textural analysis was conducted on pecan cores and intact pecans to measure the area under curve, fracturability, hardness, cohesiveness, chewiness, springiness, and *rift* ratio. It was observed that values for the *rift* ratio obtained using the intact pecan method had high *R* ^2^ (0.72) as compared to the rest of the textural attributes. A three‐parameter logistic model was employed to predict pecan texture during storage. The pecans stored at 75, 80, and 90% reached the rift ratio (F/H) of 0.5 at approx. 115, 3, and 0.15 days (~ 4 hr), respectively. Similarly, pecans stored in LDPE, PP, and PEN packs at 80% reached rift ratio (F/H) of 0.5 at approx. 26, 57, and 78 days, respectively. The presence of any kind of package delayed fracturability loss by at least eight folds at 80% RH. The pecans stored in ML did not experience a significant change in textural attributes

## Graphical abstract

The paper investigated the changes in texture of pecans stored at wide range of relative humidity (30–90%) and packaging materials (LDPE, PP, PEN, metallic laminates [ML]). This work also discusses a robust approach to instrumentally determine pecan texture by introducing *rift* (Fracturability to Hardness) ratio, which is capable of addressing variability issues in pecan texture measurements. Furthermore, three‐parameter logistic (3PL) model and predictive equations were developed which can predict the extent of changes in pecan texture during storage.

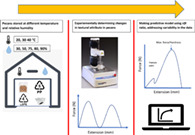

## 1 INTRODUCTION

Pecan is among the few US native crops with an annual crop value of 560–700 million USD (NASS, 2020). The Pecan trees are alternate bearing and take anywhere between 5 and 10 years before producing nuts (Zhang, Peng, & Li, 2015). The yield of pecan trees is often diminished by factors such as excessive rain, drought, winds, sunlight exposure, or damage inflicted by insects, rodents, birds, or molds (Erickson, Santerre, & Malingre, 1994). One strategy used by pecan growers and processors is to store nuts for extended periods to ensure a buffer to meet production demand both within and outside the United States (NASS, 2020). Along with Color and aroma (Kays, 1979; Prabhakar, Bock, Kerr, & Kong, 2022), pecan texture is an important indicator of pecan quality. The absence of crispiness and/or brittleness can discourage buyers from consuming pecans and might discourage them from purchasing future products (Prabhakar, Sharma, & Kong, 2020). Several researchers have investigated the effects of different conditions encountered during harvesting, storage, and transportation on the texture of pecans including drying (Shult & Brusewitz, 1998), freezing, thawing, and freeze/thaw cycles (Anzaldúa‐Morales, Brusewitz, & Junus, 1999b; Surjadinata, Brusewitz, & Bellmer, 2001), oil removal (Shult & Brusewitz, 1998; Zhang, Brusewitz, Maness, & Gasem, 1995), and moisture restoration (Anzaldua‐Morales, Brusewitz, & Maness, 1998).

Pecan kernels are non‐uniform in size and have an irregular surface structure which makes it challenging to conduct instrumental measures of texture. The asymmetrical surface makes it difficult to attain a repeatable contact area between probe and nut, thus introducing variations that cause the test method to be inaccurate (Bourne, 2002). Thus, researchers have relied on sensory panelists for texture evaluation (Ocòn, Anzaldúa‐Morales, Quintero, & Gastélum, 1995; Resurreccion & Heaton, 1987; Taipina, Lamardo, Rodas, & del Mastro, 2009). Resurreccion and Heaton (1987) developed an objective texture method for distinguishing differences between early and traditionally harvested pecans. The authors conducted the puncture test and calculated the shear force required to cut the pecan halves using a blunt blade attachment. The proposed method did not reflect situations where pecans experience mechanical deformation during handling and storage. To address issues with sample non‐uniformity, Ocòn, Anzaldúa‐Morales, Quintero, and Gastélum (1995) proposed a method where samples are prepared by driving a cork borer perpendicularly through the pecan kernel and taking out cylinders of uniform dimensions (Prabhakar, Sharma, & Kong, 2020). The core method is the most adopted textural analysis method for pecan texture (Anzaldúa‐Morales, Brusewitz, & Junus, 1999b; Shult & Brusewitz, 1998; Surjadinata, Brusewitz, & Bellmer, 2001). However, these researchers found a very low correlation between sensory and instrumental analysis for texture determination, and higher variability, indicating a need for more accurate ways to determine textural attributes.

There are many reports of the effect of processing methods (roasting, drying, dehydration, etc.) on the texture of walnuts (Kita & Figiel, 2007), pistachio (Farahnaky & Kamali, 2015; Mohammadi Moghaddam, Razavi, Taghizadeh, & Sazgarnia, 2016), macadamia (Domı, Azuara, Vernon‐Carter, & Beristain, 2007; Tu et al., 2021), pecans (Zhang et al., 2019) and pecan shells (Littlefield, Fasina, Shaw, Adhikari, & Via, 2011). However, there are few studies investigating the effects of moisture migration (from kernels to environment and vice versa) on pecan kernel texture and tree nuts in general, with and/or without the use of commonly available packaging materials such as PE, PP, cellophane, and so forth (Prabhakar et al., 2020). Furthermore, the ability to predict changes in texture under a given set of conditions is valuable for the industry to maximize the quality of kernels during storage, or to maximize shelf life once in a store for consumers. Probabilistic models can be used to predict shelf life based on a specified set of conditions. However, there is no such model(s) available for predicting changes in pecan kernel texture with changing storage/distribution conditions.

The objectives of this research were to investigate changes in pecan kernel texture due to environmental conditions (RH and packaging type) and to develop a predictive model suitable to estimate the change in the texture of pecan kernel attributes as storage progressed under different environments.

## 2 MATERIAL & METHODS

### 2.1 Pecan production, source of nutmeat, and storage experiment

Three cultivars of pecan (*Carya illinoinensis* “Stuart,” “Pawnee” and “Desirable”) were harvested from orchards located at the USDA‐Agriculture Research Service (ARS) Fruit and Tree Nut Research Laboratory, Byron, Georgia, (+32.6650 N, +83.7419 W, the elevation of ≈156 m, 240 days freeze‐free growing period, annual precipitation of 118 cm). Orchards received standard tree management practice for the state of Georgia (Wells, Prostko, & Carter, 2019). The experiment was performed twice, with pecans harvested in November 2018 and December 2019, respectively. In each season, the pecans were processed within 1 week of harvesting. The harvested pecans were conditioned before shelling by immersing in 85°C water for 3 min, followed by drying at room temperature for 20–25 min and shelling via a mechanical sheller (Modern Electronics, Mansfield, LA) (Forbus Jr & Senter, 1976). After shelling, pecans were dried at 20°C and 45% RH overnight to a moisture content of four to five percent moisture content as per AOAC *Official Method* 930.15 (AOAC INTERNATIONAL, 2012) and stored at −20°C in a commercial freezer until use in the experiments. Information on the different grades of pecans has been provided by Prabhakar, Bock, Kerr, and Kong (2022).

### 2.2 Experiment treatments

The pecans were stored in different RH conditions. The desired RH was achieved by using 200 ml saturated salt solutions placed in a Static temperature‐controlled Humidity Chamber (SHC) consisting of a 1‐L glass jar with a rubber gasket to seal the lid. More detailed information on the construction of the SHCs has been provided by Prabhakar et al. (2022). The saturated salt solutions included magnesium chloride (30–32% RH), magnesium nitrate (50–52% RH), sodium chloride (75% RH), ammonium sulfate (80–81% RH), and potassium nitrate (89–93% RH) (Certified ACS, Fisher Chemical, Waltham, MA) (Rockland, 1960). For the sake of simplicity, the RH will be denoted as 30, 50, 75, 80, and 90%, respectively. The SHCs containing pecans from three cultivars were placed in temperature‐controlled chambers at 20, 30, and 40°C. For each temperature × humidity treatment (*n* = 2 jars for each combination), 50 g of whole pecans (25–40 pecan halves) were placed in a nylon bag suspended above the saturated solutions on an aluminum mesh disc in the STC. Beaudry, Payne, and Kays (1985) showed that the different genotypes of pecans respirate and produce carbon dioxide. Since the pecans were stored in closed environment conditions (glass jars), CO_2_ is expected to accumulate and change air composition. Thus, to simulate a real storage environment and corresponding air composition, the jars were opened periodically (every 1–2 weeks) for 30 s to force fresh air into the 1‐L glass jars. In addition, some pecans were placed in packages available to pecan producers and packers viz. low‐density polypropylene (LDPE, 50–54 μm), polypropylene (PP, 45–50 μm), polyethylene‐nylon (PEN, 105–110 μm) and metallic laminates (ML, 105–110 μm). The packages were obtained from OpenTip.com and sealed using American International Electric Heat Impulse sealer (City of Industry, CA) The packaged samples were stored at 58 and 80% RH at a temperature range similar to the unpacked STC pecans. The frequency of drawing pecan samples for physical quality evaluation was based on previous studies pertaining to pecan quality changes during storage (Blackmon, 1932; Brison, 1945; Kays, 1979; Magnuson, Koppel, Reid, & Chambers IV, 2015; Mexis, Badeka, Riganakos, Karakostas, & Kontominas, 2009; Senter & Wilson, 1983). The storage time ranged from 15 to 450 days, depending on the treatment. The mold growth assessment was performed visually and samples with mold growth were discarded.

### 2.3 Sample preparation

#### 2.3.1 Pecan core method

The samples were prepared according to the method published by *Ocòn et al. (* 1995 *)*. To obtain uniform samples for texture analysis, a cork borer was inserted perpendicularly through the pecan kernels to obtain cylindrical specimens 3 mm in diameter and 5 mm in length. The cored samples were analyzed using a single compression method as the cores were not strong enough to sustain the second compression. The textural attributes studied included the first peak (hardness) and area under the curve (AUC).

#### 2.3.2 Intact pecan‐halve method

The intact pecan kernels or halves were compressed under a flat probe for texture profile analysis (TPA, double compression). The textural attributes studied included fracturability, hardness, cohesiveness, springiness, and chewiness. In TPA, these textural attributes can be defined as follow; *fracturability* is the first break in the curve force versus extension/time curve, *hardness* is the highest force on the first compression cycle (always followed by fracturability), *cohesiveness* is the ratio of (positive) first and second force areas, *springiness* is the recovery distance between the end of first and start of the second compression, chewiness is a product of hardness × cohesiveness × springiness and can be defined as the force required to chew the food product. In addition to these textural attributes, fracturability/hardness ratio (F/H, referred to as *rift ratio* from this point onwards) was also studied.

The cored and intact pecan samples were compressed up to 50% of strain under a 55 mm compression probe using a TA.XT2 texture analyzer (Texture Technologies Corporation, Scarsdale, New York/Stable Micro Systems, Haslemere, Surrey, United Kingdom). The test parameters were as follow: pre‐test speed – 1 mm/s, test speed –5 mm/s, and post‐test speed –5 mm/s. A total of 10 measurements were taken from unpacked and packed pecans. The packed pecans were only analyzed using the intact pecan method.

### 2.4 Predictive model

**FIGURE 1:**
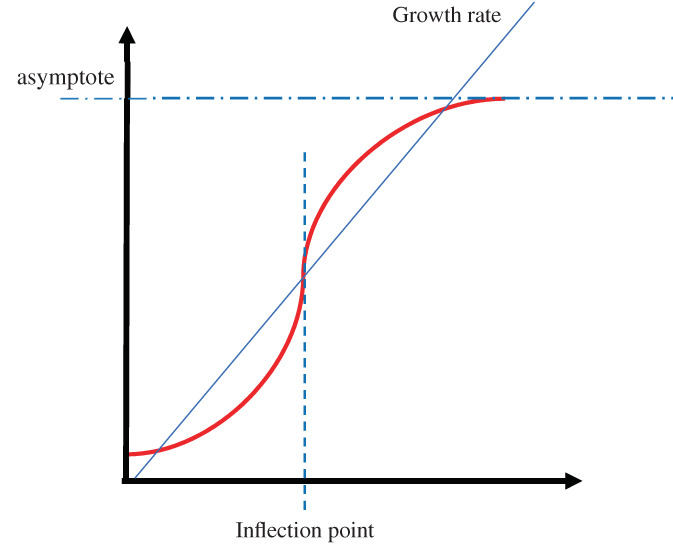
The three parameter logistic (3PL) model indicating the model parameters including the asymptote, inflection point, and growth rate. The illustration serves the purpose visualizing the model as well as its parameter and not representing any of the actual data obtained during the experimentation

A three‐parameter logistic (3PL) model, a type of sigmoid model, was used to predict the changes in pecan textural attributes over time. The 3PL model is a type of logistic model prominently used in immunoassays research (Herman, Scherer, & Shan, 2008) such as ELISA, microbial growth prediction (Fujikawa, 2010), dose–response relationships (Andrade‐Mogrovejo et al., 2022; Carøe, Ebbehøj, Bonde, Flachs, & Agner, 2018; ElHarouni et al., 2022), and geological phenomena (Chen et al., 2019; Joshuva, Deenadayalan, Sivakumar, Sathishkumar, & Vishnuvardhan, 2019). The parameters give unique information such as maximum value to response achieved (asymptote), slope, and the value of a predictor variable for median response (inflection point) (Figure 1). The 3PL model equation can be denoted as:

(1) $$\hat{y}=\frac{c}{1+\exp^{\left(-\mathrm{ax}+\mathrm{ab}\right)}}$$

where *a* is the slope, *b* is the inflection point, *c* is the asymptote and *ŷ* is the predicted response. The logistic model was built using a non‐linear function in JMP, Version 16 Pro (SAS Institute Inc., Cary, NC).

### 2.5 Experimental design and statistical analyses

The design of the experiment was a generalized randomized complete block design (GRCBD) where storage days and RH were experimental factors and cultivars were treated as a block. The whole experiment was repeated twice, indicating replication within each block. To avoid complexity and simplify the interpretation of the statistical output, interactions of block and treatment with other factors were omitted. The preliminary experiment indicated no significant effect of temperature on any of the textural attributes of pecans (*p* > .05). Thus, readings from all temperature conditions were pooled for the analysis. The outliers were determined and removed using the “jacknife distance” method.

(2) $$J_{i}=\sqrt{\frac{\left(n-2\right)n^{2}}{{\left(n-1\right)}^{3}}\times \frac{{M_{i}}^{2}}{1-\frac{nM_{i}^{2}}{{\left(n-1\right)}^{2}}}}$$

where *n* = number of observations, *p* = number of variables, and *M*_i_ = Mahalanobis distance for the *i*th observation. The upper critical line (UCL) is the limit beyond which the *J*_i_ values are considered outliers and could be omitted from the analysis. Penny (1996) has provided a detailed account on calculating UCL for jacknife analysis. Subsequently, a mixed model analysis was performed on refined data. The experimental data were normally distributed, and the model residual plots did not indicate heteroskedasticity. The model fixed effects were RH and storage days whereas cultivar was considered a random effect. The storage days were nested within RH. The dependent variables for the core method were hardness and AUC and for the intact pecan, method were fracturability, hardness, rift ratio (F/H), cohesiveness, chewiness, and springiness. The 3PL model parameters were statistically analyzed using one‐way ANOVA. The main effects and their interactions (where applicable) were studied and their interactions with blocks were omitted. The fit for the statistical models (mixed model, ANOVA, and 3PL) was assessed based on the adjusted coefficient of determination (adj. *R* ^2^). A Tukey's HSD post hoc test (confidence level, *ɑ* = 95%) was performed to explore differences among means for the different treatments. Multivariate correlation analysis was conducted to understand the relationship among multiple dependent factors (textural attributes). All statistical analyses were performed using JMP, Version 16 Pro (SAS Institute Inc., Cary, NC).

## 3 RESULTS

### 3.1 Pecan core method

**FIGURE 2:**
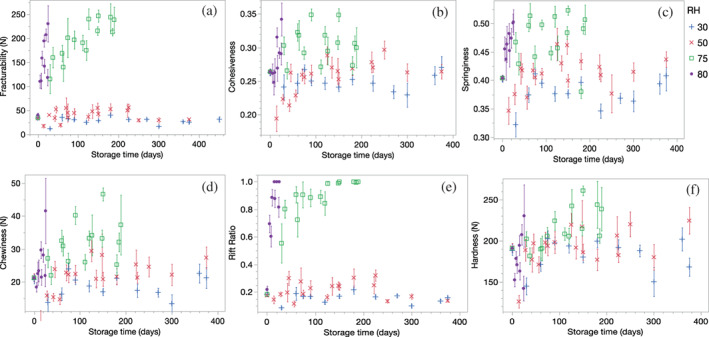
The change in textural attributes of nonpackaged pecan kernels under different relative humidities (RH) and storage duration. (a). Fracturability, (b). Cohesiveness, (c). Springiness, (d). Chewiness (N), (e). F/H (rift) ratio and (f) Hardness (N)

The change in AUC and hardness with storage time is tabulated in Table S1. The total work done significantly decreased with an increase in RH and storage time (Table 1). The change in AUC during storage was small among pecans stored between 30 and 75% RH. The change in AUC was greatest for pecans kept at 80% RH. The hardness of the cored pecans was significantly affected by RH and storage time (Table 1). The hardness value increased with greater RH. At higher humidity conditions (≧75%), the hardness increased as storage progressed. The pecans stored at and below 50% experienced a significant decrease in hardness with storage time (Figure 2). Even though the goodness of fit for AUC and hardness was low (0.15 and 0.24, respectively), the statistical significance of the main effects and interactions do indicate that the independent variables were affected by the predictors. The detailed tabulation of change in AUC and hardness with respect to RH and storage days can be found in Table S1 and Table S2.

**TABLE 1:** Mixed model analysis and means for the effects of relative humidity (RH) and storage duration (SD, days) on textural attributes of *nonpackaged pecans* (cores or kernels).

Method	Variable	Source	F ratio	Prob > F	Adj. *R* ^2^ (model)	% RH	Least Sq mean^a^		95% confidence limits
Core method	Total area under curve	SD (RH)	2.88	0.02	0.14	30	18.87	A	16.97–18.64
		RH	8.66	<0.0001		50	19.88	A	19.25–20.54
						75	17.74	B	17.17–18.30
						80	18.99	A	18.30–19.70
	Hardness (N)^**b**^	SD (RH)	7.80	<0.0001	0.24	30	15.81	C	15.21–16.41
		RH	10.06	<0.0001		50	16.06	C	15.59–16.52
						75	17.15	B	16.75–17.55
						80	19.79	A	17.9–21.68
Intact pecan‐halve method	Fracturability (N)^**b**^	SD (RH)	123.34	<0.0001	0.67	30	33.51	C	26.94–40.09
		RH	349.88	<0.0001		50	45.40	C	39.03–51.78
						75	167.92	B	160.98–174.85
						80	604.37	A	511.88–696.86
	Cohesiveness	SD (RH)	5.64	0.0002	0.12	30	0.25	B	0.24–0.26
		RH	27.94	<0.0001		50	0.26	B	0.25–0.27
						75	0.30	A	0.29–0.31
						80	0.34	C	0.32–0.36
	Springiness	SD (RH)	22.20	<0.0001	0.27	30	0.39	D	0.38–0.4
		RH	63.49	<0.0001		50	0.41	C	0.4–0.42
						75	0.47	B	0.46–0.48
						80	0.73	A	0.6–0.85
	Chewiness (N)^**b**^	SD (RH)	11.64	<0.0001	0.17	30	19.43	B	17.87–21
		RH	37.52	<0.0001		50	21.18	B	19.67–22.7
						75	30.82	A	29.18–32.47
						80	35.62	C	33.65–37.59
	F/H	SD (RH)	161.04	<0.0001	0.72	30	0.18	D	0.15–0.21
		RH	471.98	<0.0001		50	0.25	C	0.22–0.27
						7	0.80	B	0.78–0.83
						80	1.00	A	0.94–1.06

*Note*: For the analysis SD was nested within RH (SD [RH]).

^a^ Different letters for the means in each RH group indicate significant difference between the means based on Tukey's HSD (*α* = .05).

^b^ N‐newton to indicate force.

### 3.2 Intact pecan‐halve method

#### 3.2.1 Fracturability and hardness

The Fracturability of pecans was most significantly affected by RH as substantiated by the goodness of fit (*R* ^2^–0.67). The fracturability could be defined as the first break point on the TPA curve corresponding to a force value. Thus, a lower force value corresponds to high fracturability as it signals early fracturability. For unpacked pecans, the increase in RH caused the fracturability value to increase indicating the pecans were losing brittleness. The change in fracturability increased significantly as RH increased >50% (Table 1).

For pecans stored in different package materials, the fracturability significantly decreased with a change in RH, storage period, and packaging material (Table 2). The loss of fracturability was minimum in pecans stored in ML and maximum in pecans stored in LDPE packages. The loss in fracturability was intermediate in PEN and PP packages but the value was in proximity to that for samples in LDPE (TS3). The overall fracturability of pecans stored in LDPE, PP, and PEN at 58% RH was significantly lower than those stored at 80% RH. The impact of environment RH was negligible for samples stored in ML. A detailed tabulation of changes in fracturability with RH conditions can be found in TS3. Unlike results measured using the core method, the TPA of packed and unpacked pecans did not reveal a definite pattern in terms of change in hardness across storage days and RH.

**TABLE 2:** Mixed model analysis and means for the effects of relative humidity (RH) and storage duration (SD, days) on textural attributes of pecan kernels in *different types of packaging materials*.

Variable	Source	F ratio	Prob > F	Adj. *R* ^2^ (model)	Package^a^	Least sq mean^b^		95% confidence limits
Fracturability (N)^c^	Package	43.19	<0.01	0.70	AL	30.70	C	14.18–47.21
	SD (RH)	83.32	<0.01		LDPE	147.43	A	135.29–159.57
	RH	352.84	<0.01		PEN	119.49	B	108.28–130.71
	Package×RH	39.44	<0.01		PP	118.71	B	109.06–128.37
	SD×package (RH)	8.01	<0.01					
Cohesiveness	Package	5.98	<0.01	0.17	AL	0.30	B	0.28–0.31
	SD (RH)	8.36	<0.01		LDPE	0.33	A	0.32–0.34
	RH	23.501	<0.01		PEN	0.31	B	0.3–0.32
	Package×RH	10.12	<0.01		PP	0.31	B	0.3–0.32
	SD×package (RH)	2.03	0.06					
Springiness	Package	0.68	0.567	0.11	AL	0.46	A	0.44–0.49
	SD(RH)	9.92	<0.01		LDPE	0.45	A	0.44–0.47
	RH	9.15	<0.01		PP	0.47	A	0.45–0.48
	Package×*RH	1.30	0.27		PEN	0.46	A	0.45–0.47
	SD×*package (RH)	0.63	0.71					
Chewiness (N)^b^	Package	1.38	0.25	0.14	AL	31.83	A	28.03–35.64
	SD (RH)	3.61	0.03		LDPE	34.51	A	31.98–37.05
	RH	11.91	<0.01		PEN	31.99	A	29.52–34.47
	Package×RH	5.12	<0.01		PP	34.79	A	32.63–36.95
	SD×package (RH)	1.15	0.33					
F/H	Package	41.98	<0.01	0.71	AL	0.13	C	0.07–0.2
	SD (RH)	82.78	<0.01		LDPE	0.60	A	0.55–0.65
	RH	347.54	<0.01		PEN	0.53	AB	0.48–0.57
	Package×RH	33.69	<0.01		PP	0.49	B	0.45–0.53
	SD×package (RH)	8.03	<0.01					

*Note*: The least mean square indicates the textural attribute for the pecan kernels stored in different packaging at 80% RH. For the analysis SD was nested within RH (SD [RH]).

^a^ Packaging materials are LDPE, low density polypropylene, PE, polyethylene; PP, polypropylene, and ML, metal laminate.

^b^ Different letters for the means in each packaging group indicate significant difference between the means based on Tukey's HSD (*α* = .05).

^c^ N‐newton to indicate force.

#### 3.2.2 Rift ratio **(**F/H)

There were significant effects of RH and storage duration on the rift ratio (Table 1). F/H was greatest at 80% RH and least at 30% RH, and the coefficient of determination of mixed model analysis (*R* ^2^ = 0.72) further indicated a relationship between RH and storage duration on F/H. There were significant effects of packaging on F/H (Table 2). The LDPE experienced a maximum increase in *rift* ratio followed by PE, PP, and ML. The difference in the *rift* ratio of LDPE, PE, PP, and ML stored at 58% RH was not significant. This ratio was further explored for use in predictive modeling to estimate the loss of brittleness during storage. The 3PL model was employed to predict the change in *rift* ratio with storage time. The slope (change in response units per day) indicates an increase in the *rift* ratio with time, the inflection point is defined as the time taken to lose half of the fracturability value, and the asymptote refers to the maximum *rift* ratio retained during storage. In addition, the time taken for pecan to lose all fracturability at constant RH conditions can also be determined. During storage, it was observed that packaged and unpackaged pecans stored at 58% RH or below did not experience a significant loss of fracturability. Thus, a predictive model was made only for pecans stored at 75% or above.

Values of the logistic parameters for packaged and unpackaged pecans are tabulated in Table 3. The slope and inflection point for unpackaged pecans significantly increased with an increase in RH. From the *rift* point in Table 3, it was determined that unpackaged pecans stored at 75, 80, and 90% lost half of the initial *rift* ratio at ~115, 3, and 0.15 days (~ 4 hr), respectively. By multiplying the inflection point by two, the time required at constant RH for complete loss of fracturability (*rift* ratio = 1.0) was calculated as 230 days for 75% RH, 6 days for 80% RH, and 0.30 days (8 hr) for 90% RH. The slope and inflection point for RH between 75 and 90% can be determined by the following equations:

(3) $$\text{Growth rate}\left(\text{unpacked pecans}\right)=-51.24+0.70*\mathrm{RH}$$

(4) $$\text{Inflection point}\left(\text{unpacked pecans}\right)=2.72^{\left(36.16-0.43*\mathrm{RH}\right)}$$

Since no definite trend was observed for the asymptote, based on the value for nonpackaged pecans can be assumed to be 0.95 by taking the average of asymptote.

**TABLE 3:** The parameters for three‐parameter logistic model of the rift (fracturability/hardness) ratio for nonpackaged (stored at 75, 80 and 90% relative humidity (RH)) and packaged pecan kernels (80% RH)

Unpacked pecans						Packed pecans				
Slope						Slope				
RH	Least Sq mean		Lower 95%	Upper 95%	*R* ^2^	Package	Least Sq mean	Lower 95%	Upper 95%	*R* ^2^
75	1.23	C	0.34	2.12	0.90	LDPE	0.06	0.06	0.06	0.88
80	5.35	B	4.46	6.24	0.97	PEN	0.02	0.02	0.02	0.98
90	11.89	A	11.00	12.78	0.63	PP	0.03	0.03	0.03	0.98
Inflection point						Inflection point				
75	115.44	A	100.70	130.20	0.90	LDPE	26.25	26.23	26.28	0.88
80	3.17	B	−11.6	17.93	0.97	PEN	78.05	75.01	81.10	0.98
90	0.15	B	−14.6	14.90	0.63	PP	56.99	54.17	59.81	0.98
Asymptote						Asymptote				
75	0.99	A	0.88	1.09	0.90	LDPE	1.00	1.00	1.00	0.88
80	0.92	A	0.82	1.03	0.97	PEN	1.01	1.00	1.03	0.98
90	0.95	A	0.85	1.05	0.63	PP	1.01	0.99	1.02	0.98

*Note*: Different letters for each parameter group indicate significant difference between the means based (along the column) on Tukey's HSD (*α* = .05). The modeling for packed was done Pawnee only (unlike unpacked pecans with three cultivars). Thus, no ANOVA analysis was performed.

Table 3 contains the slope, inflection point, and asymptote values for packaged pecans stored at 80% RH. The slope was highest in LDPE, followed by PP and PE, indicating a higher water absorption rate in the LDPE package. The pecans stored in LDPE, PP, and PEN at 80% RH lost half of initial fracturability at ~26, 57, and 78 days, respectively. The number of days to reach complete loss of fracturability at constant RH was calculated as: LDPE – 52 days, PP – 114 days, and PEN – 156 days. The *rift* ratio for packaged pecans stored in 58% RH remained unchanged during storage. Under extreme humidity conditions, the packages provided a decent barrier against moisture transfer, delaying loss of fracturability as compared to pecans with no package, where the fracturability loss occurred in a matter of hours. The following equation predicts the *rift* ratio at a specific storage day for pecans packaged in the abovementioned materials:

(5) F/H=0.35+PC+0.0026*Storage days

where PC is a “package constant” with values as follows: LDPE = 0.084, PEN = −0.042, PP = −0.041. The water vapor transmission rate corresponding to LDPE, PEN, and PP are 1.30, 0.41, and 0.50 g.ml/24 hr100 in^2^, respectively (38°C, 50−100% RH) (Tock, 1983).

#### 3.2.3 Cohesiveness

**FIGURE 3:**
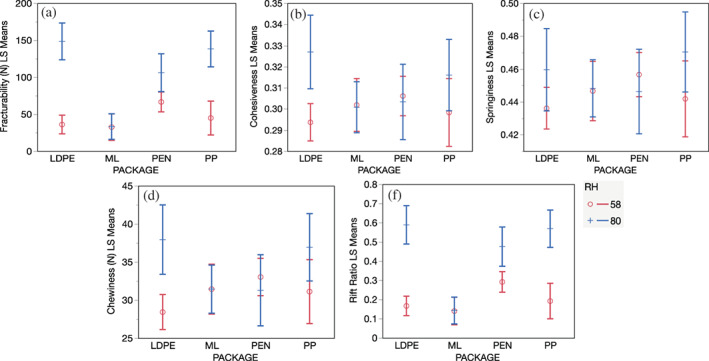
The interaction plots for change in textural attributes of pecan kernels packaged in different materials and under different relative humidities (RH) and storage durations. (a). Fracturability, (b). Cohesiveness, (c). Springiness, (d).Chewiness (N), and F. F/H (rift) ratio. Packaging materials are LDPE, low density polypropylene; PEN, polyethylene nylon; PP, polypropylene; and ML, metal laminate

The cohesiveness of unpackaged and packaged pecan kernels was significantly affected by RH and storage duration, with adjusted *R* ^2^ of 0.12 and 0.17, respectively (Table 1). The cohesiveness of unpackaged pecans significantly increased with an increase in RH. The unpackaged pecans stored at 75% RH or above experienced a sharp increase in cohesiveness (Figure 3). The pecans stored in ML had a minimum cohesiveness, however, and were not significantly different from PP and PEN (Table 2). LDPE packaged pecans experienced the greatest change in cohesiveness among all the packages.

#### 3.2.4 Springiness

The springiness of unpackaged pecans was significantly affected by RH and storage time (Table 1). The change in RH and packaging material had little to no impact on change in the springiness of packaged (<58%) and unpackaged pecans (<50%) (Table 2). The springiness increased significantly at RH levels higher than 75%. Despite exhibiting a significant effect, the adjusted *R* ^2^ for unpackaged and packed pecans were 0.27 and 0.11, respectively, indicating the limited ability of predictor variables to estimate textural attributes.

#### 3.2.5 Chewiness

Unlike springiness, the chewiness of unpackaged and packaged pecans significantly increased with an increase in RH. The unpackaged pecans stored at or below 50% were significantly less chewy as compared with pecans stored at 75% or higher. The packaging material had no significant impact on the chewiness of pecans, indicating chewiness change was similar across packaged pecans (Tables 1 and 2, TS3). As with cohesiveness and springiness, chewiness had a low adjusted *R* ^2^ of 0.17 and 0.14 for unpackaged and packaged pecans, respectively.

## 4 DISCUSSION

The fracturing of pecans is Ocòn et al. (1995) the first sensation that a consumer comes across when ingesting pecans. Given their irregular structure, one should expect variations in observations of instrumental texture analysis. The shape and size of the pecan kernel are influenced by several factors such as sunlight exposure, cultivar, or damage by insects and rodents (Sparks, 1993). To address this problem, Ocòn et al. (1995) suggested cutting cores out of pecans and cutting the ends of cores to form a cylinder with standardized dimensions. This sample preparation technique sacrifices important key textural attributes, specifically fracturability, due to the removal of the testa and the absence of numerous fracture points. The role of pecan testa in the fracturability of pecans will be explained in detail later in the manuscript. Another issue we experienced is that the pecan kernels kept at low RH (≦50%) started crumbling as the cork borer was inserted, making it difficult to maintain intact samples. As Ocòn et al. (1995) acknowledged, this sample preparation technique is also time‐consuming, making it an inconvenient protocol to follow in an extensive storage study (Anzaldúa‐Morales, Brusewitz, & Anderson, 1999a; Surjadinata et al., 2001).

**FIGURE 4:**
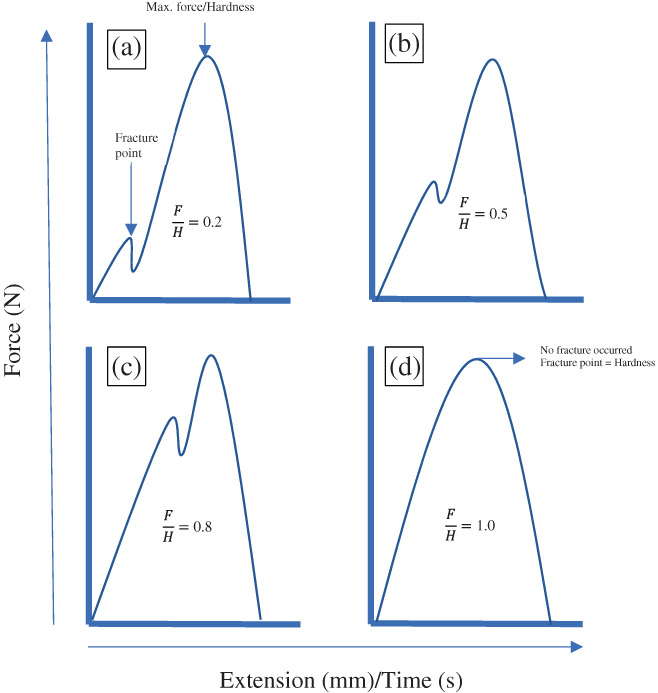
The change in the rift (F/H) ratio with constant hardness (N) during first compression. Graphs (a) to (d) represent with brittle/crisp texture to spongy/soft texture, respectively

**FIGURE 5:**
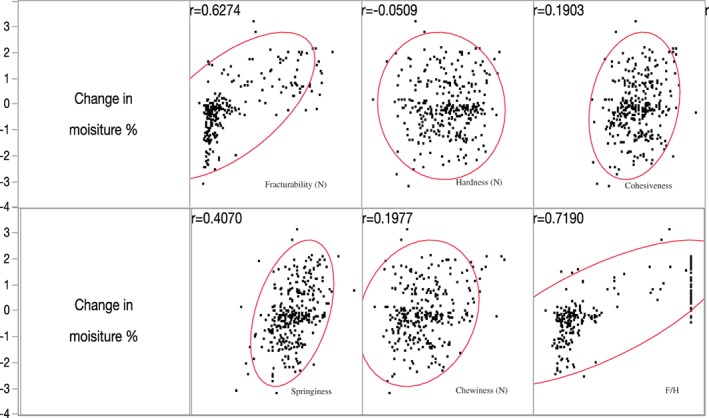
Correlation (r) between the percent change in weight and textural attributes of pecan kernels

One issue with purely compressive tests is that each pecan has a unique overall size (within a range) and an undulating surface; thus, when a disk‐shaped probe pushes through the sample it experiences differing forces based on both the material properties and the total cross‐sectional area the probe is contacting. This influences both the maximum measured force as well as the force experienced at the first fracture point if it exists. The reality of non‐uniform samples has long hampered the ability to make precise measurements of texture attributes. One‐way researchers have addressed this problem is to normalize any force‐time data by the sample volume or weight. In this work we tested the hypothesis that normalizing the “fracturability,” that is the force at first break under compression, by the force experienced under full compression, often defined as the “hardness,” would help mitigate issues with sample variability. By taking the ratio of fracturability and hardness (*rift* ratio), the onset of fracturing could be compared across multiple pecan kernels, with varying mass and size, since the ratio would take into account the maximum force experienced by the kernel during the test. The minimum value of the ratio could be zero, indicating a very brittle/crisp product (such as potato chips) while a maximum value of one would indicate no fracturability at all (such as chewy products) (Figure 4). It was statistically determined that moisture was primarily responsible for changes in the texture of pecan kernels stored under different temperatures and RH conditions. To better understand the effect of moisture on textural attributes, the moisture migration from the environment to pecans (and vice versa) were tracked using the % change in weight of pecan kernels. The multivariate analyses revealed that the *rift* ratio had a moderate positive correlation with the change in the weight of pecan kernels (Figure 5). A sigmoid model could be used to predict the F/H ratio with the change in % of the weight of pecans. A sharp increase in F/H value can be detected as the % weight of pecan kernels increases beyond 0.12%. The F/H ratio reaches 0.5 and 1.0 as the change in weight reaches 0.25 and 0.50%, respectively. The logistic model parameter for F/H and % change in weight can be found in TS4.

**FIGURE 6:**
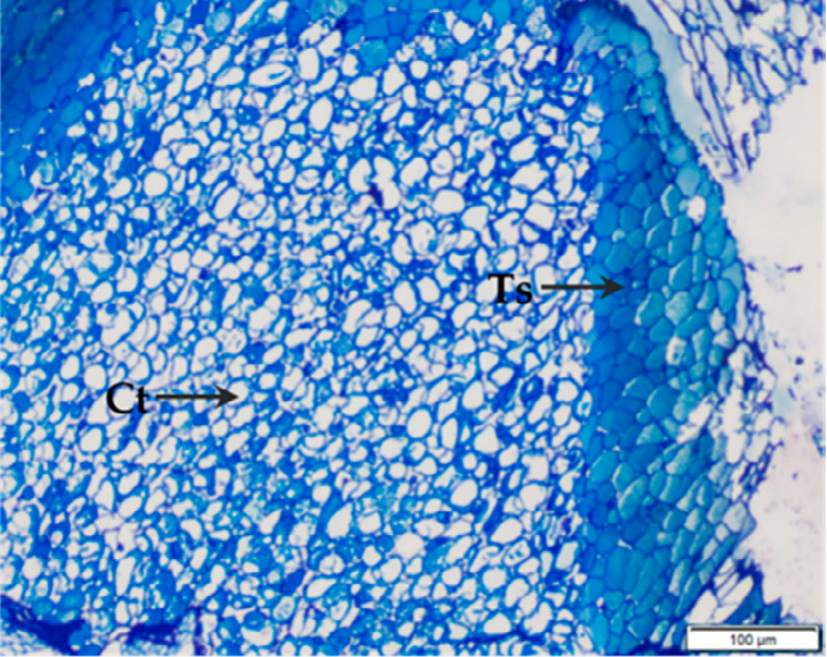
Light microscopy micrographs of the testa (Ts) and cotyledon tissue (Ct) of dry pecan nuts, adapted from Rábago‐Panduro, Morales‐de la Peña, Romero‐Fabregat, Martín‐Belloso, and Welti‐Chanes (2021)

The migration of moisture occurs due to a difference in water vapor pressure and water activity between the product and surroundings. The food products with higher water activity lead to moisture loss from product to environment increases and vice versa (Afolabi, 2014). At low moisture content, the plant cells become condensed and fragile, contributing to brittleness and easy fracturability (Capuano, Pellegrini, Ntone, & Nikiforidis, 2018; Nikiforidis, Kiosseoglou, & Scholten, 2013). Light micrographs revealed cells in the pecan testa are much more compact than in cotyledon tissues (the white meat of the pecan kernel), contributing to the brittleness of pecans (Figure 6). Additionally, testa is present is the barrier between cotyledon and environment. Such an arrangement of cells make pecan testa more susceptible to moisture absorption (Rábago‐Panduro, Morales‐de la Peña, Romero‐Fabregat, Martín‐Belloso, & Welti‐Chanes, 2021). As moisture in the pecan increases, the compact cells start to swell and have greater cell wall flexibility and an increase in the intracellular distance, causing loss of fracturability. The increase in the concentration of water molecules and the presence of oleosomes (oil storage entities in pecans) contributes to a cushioning effect against compressive forces and increases springiness (Capuano, Pellegrini, Ntone, & Nikiforidis, 2018). As the moisture increased during storage, the pecans became more cohesive, indicating resistance toward a breakdown. In addition, the overall kernel mass increased, which contributed to cohesiveness due to new hydrogen bond formation (Blahovec, 2007). This would also indicate that the pecan with increased moisture levels required more work to chew, which was indeed indicated by an increase in chewiness.

For samples placed in any packaging, the fracturability loss was delayed by at least 8‐fold (Table 3). For packaged pecans exposed to 80% RH, kernels stored in LDPE experienced a greater gain in moisture than those in PP, PEN, and laminate because of the greater water vapor transmission rate (Tock, 1983). That is, the WVTR for LDPE was 1.30 g. ml/24 hr100 in^2^ (38°C, 50–100% RH), compared to values of 0.50 and 0.41 g.ml/24 hr100 in^2^ for PP and PEN, respectively. Unlike LDPE, PEN, and PP, the metal laminate package was essentially impervious to moisture migration. As moisture could not enter, the textural attributes of pecans did not change significantly in the laminate packages, making them suitable packaging material for pecan kernels being handled in a high RH environment.

## 5 CONCLUSION

Pecan texture is one of the important quality attributes of pecans, along with color and flavor, that is affected by storage, handling, and distribution conditions. This study investigated the two different texture methods, viz. the core method by Ocòn et al. (1995) and compression of intact pecans, for their versatility for studying the texture of pecans in differing packages and environmental conditions. The intact pecan method was found to be a reliable indicator of texture changes when analyzed using the *rift* ratio, that is by measuring the fracture force normalized by the maximum force experienced in compression. This helped reduce some of the variability of the data. Out of all the textural attributes, fracturability was found to be the most sensitive indicator in terms of reacting to environmental moisture content. Pecans became less fracturable and more cohesive, chewy, and springy as moisture migrated from the environment into pecans. Fracturability was drastically reduced as the environment RH was >50% for unpackaged pecans and >58% for packaged pecans. It was found that any kind of moisture barrier around pecans was able to deter texture change by at least 8‐fold. Pecans kept in LDPE experienced the greatest change in texture whereas pecans in ML did not change significantly during the storage. For the first time, a model and predictive equations were built to estimate changes in textural attributes of pecans along with meaningful model parameters such as slope and inflection point. Thus, our study explores the possibility of integration of stochastic models from other fields of STEM into food science research to build consequential models able to predict texture change in food.

## AUTHOR CONTRIBUTIONS

Himanshu Prabhakar: Design of experiment, data collection, statistical analysis, writing. Clive H. Bock: statistical analysis, supervision, writing, and editing. William L. Kerr: instrumental analysis, supervision, writing, and editing. Fanbin Kong: Conceptualization and design of experimental, supervision, writing, and editing.

## ETHICAL STATEMENTS

Conflict of Interest: The authors declare no competing interests.

Ethical Review: This study does not involve any human or animal testing.

## Supporting information


**Supplementary Table S1** Total area under the curve (AUC) for work done to compress cores of nonpackaged pecan kernels subject to compression tests after being stored under different storage duration and relative humidity (RH) conditions.
Supplementary Table T2 Hardness of cores of nonpackaged pecan kernels subject to compression tests after being stored under different storage duration and relative humidity (RH) conditions.
Supplementary Table T3 Summary of textural attributes of pecan kernels subject to compression tests after being stored in different packaging under different storage duration and relative humidity (RH) conditions.
Supplementary Table T4 Summary of the parameters for the three‐parameter logistic model of the rift (fracturability/hardness) ratio versus the % change in weight of unpacked pecan kernels (*R* ^2^–0.73).

## Data Availability

The data that support the findings of this study are available from the corresponding author upon reasonable request.
