# Supplementary material for: Effect of relative humidity, storage days, and packaging on pecan kernel texture: Analyses and modeling
Source: J Texture Stud. 2022 Oct 3;54(1):115–26. doi: 10.1111/jtxs.12723 (PMC10092868; doi:10.1111/jtxs.12723)
Supplement: Supplementary file 1 — Supplementary Table S1 Total area under the curve (AUC) for work done to compress cores of nonpackaged pecan kernels subject to compression tests after being stored under different storage duration and relative humidity (RH) conditions. Supplementary Table T2 Hardness of cores of nonpackaged pecan kernels subject to compression tests after being stored under different storage duration and relative humidity (RH) conditions. Supplementary Table T3 Summary of textural attributes of pecan kernels subject to compression tests after being stored in different packaging under different storage duration and relative humidity (RH) conditions. Supplementary Table T4 Summary of the parameters for the three‐parameter logistic model of the rift (fracturability/hardness) ratio versus the % change in weight of unpacked pecan kernels (R 2–0.73). [file JTXS-54-115-s001.docx]

**EFFECT OF RELATIVE HUMIDITY, STORAGE DAYS AND PACKAGING ON PECAN KERNEL TEXTURE: ANALYSES AND MODELING**

Himanshu Prabhakar^a^, William L. Kerr^a^, Clive H. Bock^b^, Fanbin Kong^a*^

^a^ Department of Food Science & Technology, University of Georgia, Athens, GA, US.

^b^ USDA-ARS-SEFNTRL, Byron, GA, USA

*Corresponding author: fkong@uga.edu

Supplementary Table T1 Total area under the curve (AUC) for work done to compress cores of nonpackaged pecan kernels subject to compression tests after being stored under different storage duration and relative humidity (RH) conditions

| **RH** | **Storage Days** | **LSmean (AUC)** ^a^ |  | **95% Confidence limits** |
| --- | --- | --- | --- | --- |
| 30 | 0 | 20.39 | ABC | 18.83-21.96 |
|  | 30 | 17.19 | ABCD | 15.13-19.24 |
|  | 60 | 18.99 | ABCD | 17.27-20.72 |
|  | 75 | 19.49 | ABCD | 17.79-21.18 |
|  | 90 | 17.30 | ABCD | 15.91-18.68 |
|  | 120 | 19.02 | ABCD | 17.26-20.78 |
|  | 150 | 18.50 | ABCD | 17.06-19.94 |
|  | 180 | 21.07 | ABCD | 18.49-23.66 |
|  | 225 | 18.20 | ABCD | 15.03-21.37 |
| 50 | 0 | 19.45 | ABCD | 17.62-21.28 |
|  | 14 | 22.16 | A | 20.04-24.27 |
|  | 28 | 21.65 | A | 19.7-23.61 |
|  | 42 | 21.72 | A | 19.61-23.84 |
|  | 45 | 19.36 | ABCD | 17.25-21.47 |
|  | 56 | 17.01 | ABCD | 14.84-19.19 |
|  | 70 | 19.60 | ABCD | 17.54-21.66 |
|  | 75 | 20.60 | ABCD | 18.54-22.66 |
|  | 90 | 21.43 | AB | 19.32-23.55 |
|  | 135 | 15.24 | ABCD | 12.4-18.07 |
|  | 180 | 15.91 | ABCD | 12.92-18.9 |
|  | 225 | 17.56 | ABCD | 15.5-19.62 |
|  | 300 | 20.46 | ABCD | 16.45-24.47 |
| 75 | 0 | 19.45 | ABCD | 17.62-21.28 |
|  | 7 | 18.74 | ABCD | 16.56-20.91 |
|  | 14 | 17.61 | ABCD | 15.56-19.67 |
|  | 21 | 18.37 | ABCD | 16.31-20.42 |
|  | 28 | 18.60 | ABCD | 16.54-20.66 |
|  | 30 | 16.95 | ABCD | 14.9-19.01 |
|  | 35 | 15.50 | BCD | 13.44-17.56 |
|  | 37 | 19.51 | ABCD | 17.46-21.57 |
|  | 60 | 17.47 | ABCD | 15.46-19.48 |
|  | 74 | 18.29 | ABCD | 16.28-20.29 |
|  | 90 | 15.45 | BCD | 13.34-17.57 |
|  | 111 | 19.28 | ABCD | 17.17-21.4 |
|  | 120 | 19.69 | ABCD | 17.57-21.8 |
|  | 148 | 17.83 | ABCD | 15.59-20.08 |
|  | 150 | 16.99 | ABCD | 14.15-19.82 |
|  | 185 | 15.21 | ABCD | 12.22-18.2 |
| 80 | 0 | 19.44 | ABCD | 17.43-21.44 |
|  | 5 | 17.87 | ABCD | 15.87-19.88 |
|  | 7 | 21.73 | ABCD | 18.74-24.72 |
|  | 8 | 17.54 | ABCD | 14.55-20.53 |
|  | 10 | 17.92 | ABCD | 15.81-20.03 |
|  | 14 | 17.34 | ABCD | 15.33-19.34 |
|  | 15 | 19.82 | ABCD | 17.81-21.82 |
|  | 16 | 21.27 | ABCD | 18.28-24.26 |
|  | 21 | 14.77 | D | 12.76-16.77 |
|  | 24 | 19.72 | ABCD | 16.88-22.55 |
|  | 25 | 17.87 | ABCD | 14.88-20.86 |
|  | 28 | 15.22 | CD | 13.11-17.34 |
|  | 32 | 19.37 | ABCD | 16.53-22.21 |
|  | 35 | 17.51 | ABCD | 15.46-19.57 |
|  | 40 | 24.90 | A | 20.89-28.91 |

^a^_Different letters for the means in each RH group indicate significant difference between the means based on Tukey's HSD (α = 0.05)._

Supplementary Table T2 Hardness of cores of nonpackaged pecan kernels subject to compression tests after being stored under different storage duration and relative humidity (RH) conditions

| **RH** | **Storage Days** | **LSmean** ^a^ |  | **95% Confidence limits** |
| --- | --- | --- | --- | --- |
| 30 | 0 | 18.52 | ABCH | 17.45-19.58 |
|  | 30 | 14.72 | DEFG | 13.22-16.23 |
|  | 60 | 16.00 | BCDEFGH | 14.81-17.19 |
|  | 75 | 16.08 | BCDEFGH | 14.89-17.27 |
|  | 90 | 14.68 | E | 13.71-15.66 |
|  | 120 | 16.49 | BCDEFGH | 15.33-17.66 |
|  | 150 | 15.77 | BCDEFGH | 14.71-16.84 |
|  | 180 | 16.57 | ABCDEFGH | 14.86-18.28 |
|  | 225 | 13.49 | DEFGH | 11.23-15.75 |
| 50 | 0 | 18.93 | ABC | 17.62-20.23 |
|  | 14 | 17.45 | ABCDEFGH | 15.94-18.96 |
|  | 28 | 16.91 | ABCDEFGH | 15.52-18.31 |
|  | 42 | 16.12 | BCDEFGH | 14.61-17.62 |
|  | 45 | 14.55 | EFG | 13.09-16.02 |
|  | 56 | 14.61 | EFG | 13.15-16.08 |
|  | 70 | 15.10 | BCDEFGH | 13.63-16.57 |
|  | 75 | 16.06 | BCDEFGH | 14.55-17.57 |
|  | 90 | 16.32 | BCDEFGH | 14.89-17.75 |
|  | 135 | 13.52 | EG | 11.5-15.54 |
|  | 180 | 13.80 | CDEFGH | 11.54-16.06 |
|  | 225 | 14.24 | E | 12.81-15.67 |
|  | 300 | 18.53 | ABCDEFGH | 15.67-21.39 |
| 75 | 0 | 18.93 | ABC | 17.62-20.23 |
|  | 7 | 17.07 | ABCDEFGH | 15.47-18.67 |
|  | 14 | 15.57 | BCDEFGH | 14.06-17.07 |
|  | 21 | 16.30 | BCDEFGH | 14.87-17.73 |
|  | 28 | 17.19 | ABCDEFGH | 15.76-18.62 |
|  | 30 | 15.11 | BCDEFGH | 13.51-16.71 |
|  | 35 | 17.05 | ABCDEFGH | 15.58-18.51 |
|  | 37 | 17.76 | ABCDEFGH | 16.21-19.31 |
|  | 60 | 15.35 | BCDEFGH | 13.84-16.86 |
|  | 74 | 16.94 | ABCDEFGH | 15.51-18.37 |
|  | 90 | 17.00 | ABCDEFGH | 15.5-18.51 |
|  | 111 | 18.52 | ABCDFGH | 17.02-20.03 |
|  | 120 | 19.29 | ABC | 17.83-20.76 |
|  | 148 | 16.60 | ABCDEFGH | 15.1-18.11 |
|  | 150 | 17.76 | ABCDEFGH | 15.74-19.78 |
|  | 185 | 17.74 | ABCDEFGH | 15.72-19.76 |
| 80 | 0 | 18.69 | ABCDFH | 17.29-20.08 |
|  | 5 | 18.19 | ABCDEFGH | 16.76-19.61 |
|  | 7 | 20.36 | AB | 18.23-22.49 |
|  | 8 | 18.05 | ABCDEFGH | 16.03-20.07 |
|  | 10 | 18.16 | ABCDEFGH | 16.56-19.76 |
|  | 14 | 17.34 | ABCDEFGH | 15.91-18.77 |
|  | 15 | 19.09 | ABC | 17.63-20.56 |
|  | 16 | 20.03 | ABC | 18-22.05 |
|  | 21 | 17.14 | ABCDEFGH | 15.71-18.57 |
|  | 24 | 18.39 | ABCDEFGH | 16.37-20.41 |
|  | 25 | 18.84 | ABCDEFGH | 16.82-20.86 |
|  | 28 | 18.57 | ABCDFGH | 17.1-20.03 |
|  | 32 | 19.50 | ABCDEFGH | 17.37-21.63 |
|  | 35 | 19.08 | ABCDH | 17.53-20.63 |
|  | 40 | 23.66 | A | 20.46-26.85 |

^a^_Different letters for the means in each RH group indicate significant difference between the means based on Tukey's HSD (α = 0.05)._

Supplementary Table T3 Summary of textural attributes of pecan kernels subject to compression tests after being stored in different packaging under different storage duration and relative humidity (RH) conditions.

| **Variable** | **Package^a^** | **% RH** | **LSmean**^b^ |  | | **95% Confidence limits** |  |  |
| --- | --- | --- | --- | --- | --- | --- | --- | --- |
| **F/H** | **ML** | 58 | 0.13 | | C | 0.04-0.23 | | |
|  |  | 80 | 0.12 | | C | 0.03-0.22 | | |
|  | **LDPE** | 58 | 0.17 | | C | 0.12-0.21 | | |
|  |  | 80 | 1.02 | | A | 0.94-1.11 | | |
|  | **PEN** | 58 | 0.26 | | C | 0.22-0.31 | | |
|  |  | 80 | 0.79 | | B | 0.71-0.87 | | |
|  | **PP** | 58 | 0.20 | | C | 0.15-0.25 | | |
|  |  | 80 | 0.78 | | B | 0.72-0.84 | | |
| **Fracturability (N)** | **ML** | 58 | 30.70 | | CD | 7.34-54.05 | | |
|  |  | 80 | 30.70 | | CD | 7.34-54.05 | | |
|  | **LDPE** | 58 | 36.28 | | D | 25.33-47.23 | | |
|  |  | 80 | 258.58 | | A | 236.9-280.26 | | |
|  | **PEN** | 58 | 61.57 | | C | 50.26-72.88 | | |
|  |  | 80 | 177.42 | | B | 158.04-196.8 | | |
|  | **PP** | 58 | 46.31 | | CD | 33.93-58.7 | | |
|  |  | 80 | 191.11 | | B | 176.3-205.93 | | |
| **Cohesiveness** | **ML** | 58 | 0.30 | | BC | 0.28-0.32 | | |
|  |  | 80 | 0.30 | | BC | 0.28-0.32 | | |
|  | **LDPE** | 58 | 0.29 | | C | 0.28-0.3 | | |
|  |  | 80 | 0.37 | | A | 0.35-0.39 | | |
|  | **PEN** | 58 | 0.30 | | BC | 0.29-0.31 | | |
|  |  | 80 | 0.31 | | BC | 0.29-0.33 | | |
|  | **PP** | 58 | 0.30 | | C | 0.29-0.31 | | |
|  |  | 80 | 0.32 | | B | 0.31-0.34 | | |
| **Springiness** | **ML** | 58 | 0.46 | | AB | 0.43-0.49 | | |
|  |  | 80 | 0.46 | | AB | 0.43-0.49 | | |
|  | **LDPE** | 58 | 0.44 | | B | 0.42-0.45 | | |
|  |  | 80 | 0.47 | | AB | 0.44-0.49 | | |
|  | **PEN** | 58 | 0.45 | | B | 0.44-0.46 | | |
|  |  | 80 | 0.47 | | AB | 0.45-0.49 | | |
|  | **PP** | 58 | 0.44 | | B | 0.43-0.46 | | |
|  |  | 80 | 0.49 | | A | 0.47-0.51 | | |
| **Chewiness (N)** | **ML** | 58 | 31.83 | | AB | 26.46-37.21 | | |
|  |  | 80 | 31.83 | | AB | 26.46-37.21 | | |
|  | **LDPE** | 58 | 28.63 | | B | 26.26-31 | | |
|  |  | 80 | 40.40 | | A | 35.91-44.89 | | |
|  | **PEN** | 58 | 32.24 | | B | 29.72-34.77 | | |
|  |  | 80 | 31.74 | | AB | 27.49-36 | | |
|  | **PP** | 58 | 30.55 | | B | 27.72-33.38 | | |
|  |  | 80 | 39.04 | | A | 35.77-42.3 | | |

^a^ Packaging materials are LDPE = low density polypropylene, PEN = polyethylene nylon, PP = polypropylene, and ML = metallic laminate.

^b^ Different letters for the means in each RH group indicate significant difference between the means based on Tukey's HSD (α = 0.05).

Supplementary Table T4 Summary of the parameters for the three-parameter logistic model of the rift (fracturability/hardness) ratio vs the % change in weight of unpacked pecan kernels (R^2^ – 0.73)

| **Parameter** | **Estimate** | **95% Confidence limits** |
| --- | --- | --- |
| Slope | 6.52 | 3.89-9.15 |
| Inflection Point | 0.24 | 0.16-0.32 |
| Asymptote | 0.90 | 0.82-0.98 |
|  |  |  |
